# An Explainable Supervised Machine Learning Model for Predicting Respiratory Toxicity of Chemicals Using Optimal Molecular Descriptors

**DOI:** 10.3390/pharmaceutics14040832

**Published:** 2022-04-11

**Authors:** Keerthana Jaganathan, Hilal Tayara, Kil To Chong

**Affiliations:** 1 Department of Electronics and Information Engineering, Jeonbuk National University, Jeonju 54896, Korea; keerthanairtt@gmail.com; 2 School of International Engineering and Science, Jeonbuk National University, Jeonju 54896, Korea; 3 Advances Electronics and Information Research Center, Jeonbuk National University, Jeonju 54896, Korea

**Keywords:** respiratory toxicity, molecular descriptors, feature selection, machine learning, SHapley Additive exPlanations

## Abstract

Respiratory toxicity is a serious public health concern caused by the adverse effects of drugs or chemicals, so the pharmaceutical and chemical industries demand reliable and precise computational tools to assess the respiratory toxicity of compounds. The purpose of this study is to develop quantitative structure-activity relationship models for a large dataset of chemical compounds associated with respiratory system toxicity. First, several feature selection techniques are explored to find the optimal subset of molecular descriptors for efficient modeling. Then, eight different machine learning algorithms are utilized to construct respiratory toxicity prediction models. The support vector machine classifier outperforms all other optimized models in 10-fold cross-validation. Additionally, it outperforms the prior study by 2% in prediction accuracy and 4% in MCC. The best SVM model achieves a prediction accuracy of 86.2% and a MCC of 0.722 on the test set. The proposed SVM model predictions are explained using the SHapley Additive exPlanations approach, which prioritizes the relevance of key modeling descriptors influencing the prediction of respiratory toxicity. Thus, our proposed model would be incredibly beneficial in the early stages of drug development for predicting and understanding potential respiratory toxic compounds.

## 1. Introduction

Drug toxicity and safety evaluation studies are significant issues in the pharmaceutical industry, frequently impeding the commercialization of new drug candidates [1]. Despite preclinical assessments, drug failure is primarily due to high toxicity, accounting for two-thirds of post-approval drug withdrawals and one-fifth of clinical trial drug failures [2]. Among the concerns regarding drug toxicity, chemical-induced respiratory toxicity is a significant contributor to drug failure due to clinically significant adverse drug reactions [3,4]. Respiratory toxicity can have a significant influence on human health and can even result in death. The most frequently seen clinical signs are asthma, bronchitis, rhinitis, pneumonia, and wheezing [5,6,7,8]. In general, the adverse effects of common medications on the human respiratory system are not apparent in the initial stages [9]. Thus, it is critical to establish methods for evaluating the potential respiratory toxicity of chemicals as early as possible in the drug development process. The most concerning endpoints among the different types of drug-induced respiratory injury issues are respiratory sensitization and interstitial lung disease [10,11]. The most commonly found drugs that cause respiratory toxicity include anticancer agents, antibiotics, immunosuppressive agents, and cardiac medicines [12,13]. Cytotoxic medications such as bleomycin, cyclophosphamide, and methotrexate, as well as non-cytotoxic drugs including nitrofurantoin and amiodarone, have the potential to be toxic to the lungs [14,15,16].

Earlier, the respiratory toxicity of chemicals was investigated using a variety of animal and non-animal experimental techniques. However, these conventional experiments are expensive and time-consuming. Considering animal welfare and cost savings, a growing number of alternative toxicity evaluation methodologies have been implemented [17,18]. In comparison to experimental procedures, computational methods enable the rapid and cost-effective identification of potential respiratory toxicants from a list of chemicals. Among the several computational techniques, quantitative structure-activity relationship (QSAR) models have been extensively utilized to evaluate the toxicity of chemicals [19,20]. Numerous machine learning techniques, including support vector machines, artificial neural networks, and decision trees have been employed to develop QSAR models for predicting the toxicity of novel compounds [21,22].

Even though many QSAR models have been developed to predict chemical respiratory toxicity, the vast majority of them were developed using a small number of chemical compounds with respiratory sensitization as the primary endpoint [23,24,25]. As a result, the applicability domain of such models has been constrained, because adverse effects of chemicals on the respiratory system have additional symptoms, including bronchitis, rhinitis, or pneumonia [26,27,28]. Lei et al. [26] developed classification models for respiratory toxicity using six machine learning algorithms on a dataset of 1403 compounds. The best prediction model for the test set had an MCC of 0.644 and a global accuracy of 82.62%. Zhang et al. [27] constructed respiratory toxicity prediction models based on 1241 compounds using Naive Bayes classifiers. The best Naive Bayes model gave an overall prediction accuracy of 84.3% for the external test set. Wang et al. [28] recently employed six machine learning approaches with molecular fingerprints to build prediction models for chemical respiratory toxicity. The best RF model with the PubChem fingerprint obtained a prediction accuracy of 85.9%. Although previous machine learning models had good prediction accuracy, their practical application was limited due to the lack of explainable predictions.

Apart from model performance, model explainability is a significant criterion for the implementation of computational methods in pharmaceutical research [29,30,31]. Certain intrinsically explainable models, for example, linear models and basic decision trees, are not powerful. On the other hand, complex models such as support vector machines and artificial neural networks are highly successful yet difficult to explain [32,33]. Various explanations for black-box prediction models have been presented in the literature. In general, these explanation methods can be categorized as the model and instance explanation approaches. Model-based and instance-based explanations are also known as global and local explanations, respectively. Additionally, these explanation approaches can be model-specific or model-independent (agnostic). Unlike model-specific explanation methods, model-agnostic explanation methods can be applied to any ML model and are typically applied post-hoc [34,35]. The well-known Shapely additive explanations [36] (SHAP) algorithm can be used to provide global explanations for chemical classification and molecular activity prediction of any machine learning model [37].

**Figure 1:**
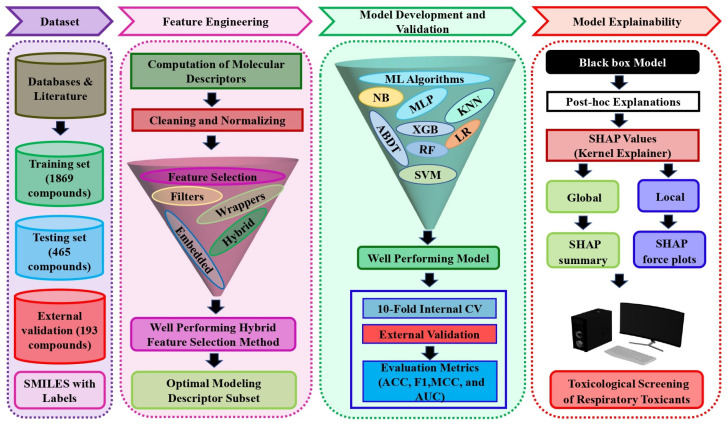
The schematic workflow of the proposed explainable respiratory toxicity prediction model.

In this paper, an extensive respiratory toxicity dataset containing 2527 compounds was used to develop predictive models using machine learning algorithms. Initially, several feature selection approaches were examined to identify significant molecular descriptors associated with respiratory toxicity. Then, a hybrid feature selection method that combines the best of the filter and wrapper methods was employed to select the optimal subset of descriptors. The classification models were built with the selected descriptors using eight different machine learning methods. The internal 10-fold cross-validation procedure was used to compare the prediction performance of the optimized models. Additionally, a test set and an external validation set were used to evaluate the reliability of the best performing SVM model. Finally, the SHAP method was used to explain the proposed black box model predictions at both local and global levels to prioritize the importance of key input molecular descriptors that influence the chemical respiratory toxicity prediction results. Figure 1 depicts the proposed explainable respiratory toxicity prediction model’s schematic workflow.

## 2. Materials and Methods

### 2.1. Dataset Preparation

The chemical substances related to respiratory system toxicity were identified through a review of the literature. Wang et al. [28] compiled a list of chemical compounds known to be hazardous to the respiratory system from the repositories PNEUMOTOX [38], ADReCS [3], and Hazardous Chemical Information System [39], as well as from pertinent literature [24,40]. Respiratory toxicants are chemicals that have negative impacts on the human respiratory system, while respiratory non-toxicants are substances that are not harmful to the respiratory system. In this study, the respiratory non-toxicants comprise both respiratory non-sensitizers and human skin non-sensitizers, as skin non-sensitizers do not cause respiratory sensitization [28,40]. First, each compound’s chemical structure was thoroughly studied and compared to a standardized simplified molecular-input line-entry system (SMILES) in the ChemIDplus database. Following that, metals, inorganic chemicals, salts, mixtures, and duplicate compounds were removed. Finally, the compounds were randomly split into the training and test sets in an 8:2 ratio. To further validate the model’s generalizability, an external validation set was obtained from previous research [28] that incorporated chemicals from the SIDER [41] and IntSide [42] databases and literature [43]. A detailed description of the list of chemicals used in this study is given in Table 1.

### 2.2. Computation of Molecular Descriptors

Molecular descriptors were used to quantitatively represent the properties of molecules [44]. The open-source web-based platform ChemDes was used to compute various classes of PaDEL molecular descriptors for each molecule used in this work [45]. This descriptor set includes physiochemical properties as well as structural one- and two-dimensional descriptors (Table 2). The list of molecular descriptors for the training dataset, test dataset, and external validation set compounds can be found in Supplementary Tables S1–S3.

### 2.3. Data Preprocessing and Feature Selection Methods

Data preprocessing and feature selection are critical steps in efficiently processing high-dimensional feature space while discarding noisy, non-informative, and redundant data to reduce computing time, improve learning accuracy, and provide a better understanding of machine learning models and features [46]. In the first step, we dropped the empty or null-valued descriptors from the original descriptor set followed by a feature scaling technique as the range of features affects the prediction performance of distance-based algorithms (KNN and SVM), whereas tree-based algorithms (RF and XGB) are relatively insensitive to feature scaling [47,48]. Finally, the selection of optimized descriptors is essential for respiratory toxicity prediction model development using the training set.

In general, feature selection approaches can be classified into three broad categories based on their mechanisms of selection: filters, wrappers, and embedding methods. Filter techniques play a prominent part in feature selection, as they can be integrated with any machine learning model and are also computationally inexpensive in the case of high-dimensional feature spaces [49]. Filter approaches assess the goodness of feature subsets solely on the basis of their essential statistical properties, by comparing a single feature or collection of features to the class label. In this investigation, we used basic multivariate filters to eliminate constant, duplicated, and almost zero variant descriptors. Additionally, correlation filtering methods were used to eliminate redundant descriptors with a high degree of mutual correlation between them and irrelevant descriptors with a very low correlation with the respiratory toxicity class [50]. The Pearson’s linear correlation coefficient, Spearman’s and Kendall’s rank correlation coefficients (nonlinear methods), were used to determine the correlation coefficient [51].

Wrapper methods were used to find the optimal subset of descriptors for the specified machine learning algorithm. To automatically select and cross-validate the optimal subset of descriptors, we employed logistic regression (LR), support vector machine (SVM), and random forest (RF) algorithms in conjunction with a recursive feature elimination search strategy [52]. In this investigation, the significant features were also selected using embedded tree-based (RF and XGB) feature selection methods based on their trained model’s feature importance. Additionally, we studied another embedded feature selection approach that relies on regularization. To select descriptors with non-zero coefficients, LASSO regularization (L1) can be used for linear classifiers (LR and Linear SVC). The performance evaluation metrics (classification accuracy, F1-score, and MCC) of the SVM classifier on 10-fold cross-validation (CV) and feature reduction rate were used to compare the prediction performance of individual feature selection techniques. Finally, various hybrid techniques for feature selection [53] (combing filters with wrappers and filters with embedded methods) were developed and analyzed to determine the final optimum feature subset.

### 2.4. Model Development and Optimization

Machine learning techniques were used to develop classification models for the prediction of chemical-induced respiratory toxicity [24,25]. Eight machine learning models were used to build the models in this study: support vector machine (SVM) [54], multi-layer perceptron (MLP) [55], extreme gradient boosting (XGB) [56], random forest (RF) [57], logistic regression (LR) [58], adaptive boosting decision tree (ABDT) [59], k-nearest neighbors (KNN) [60], and naive Bayes (NB) [61]. The above machine learning methods were implemented using Python libraries, including scikit-learn [62] and XGBoost [63]. The grid search optimization method was used to determine the optimal parameters of the models.

SVM is a well-known supervised machine learning technique used in QSAR modeling in the drug discovery field [64]. The purpose of SVM is to determine the optimum separating hyperplane that maximizes the sum of the shortest distances between data points and the hyperplane. The hyperparameters of the SVM can be tuned to avoid overfitting. The regularization parameter (C) denotes the error penalty, which regulates the trade-off between correct classification and a more smooth decision boundary. The linearity or nonlinearity of the hyperplane is determined by the kernel parameter. The kernel width (gamma) parameter is used with non-linear kernels. A brief description of the other models compared with the SVM classifier along with their optimal parameter values is given in Supplementary Table S4.

### 2.5. Model Validation and Evaluation Measures

Internal and external validations were used in this work to evaluate the developed model’s predictability and reliability. Internal validation was performed by tenfold cross-validation (CV), and external validation was accomplished using predictions from the test set and external validation set. Based on true positives (A), true negatives (D), false negatives (B), and false positives (C), each classifier was evaluated using the following parameters: accuracy (ACC), sensitivity (SEN) or recall, specificity (SPE), F1-score, and Matthews correlation coefficient (MCC) [65].

(1) $$\mathrm{Accuracy}\;\left(\mathrm{ACC}\right)=\frac{\mathrm{TP}\;+\;\mathrm{TN}}{\mathrm{TP}\;+\;\mathrm{TN}\;+\;\mathrm{FN}\;+\;\mathrm{FP}}$$

(2) $$\mathrm{Sensitivity}\;\left(\mathrm{SEN}\right)\;\mathrm{or}\;\mathrm{Recall}=\frac{\mathrm{TP}}{\mathrm{TP}\;+\;\mathrm{FN}}$$

(3) $$\mathrm{Specificity}\;\left(\mathrm{SPE}\right)=\frac{\mathrm{TN}}{\mathrm{TN}\;+\;\mathrm{FP}}$$

(4) $$F1\text{-}\mathrm{Score}=\frac{2\;\times \;\mathrm{TP}}{(2\;\times \;\mathrm{TP})+\mathrm{FN}\;+\;\mathrm{FP}}$$

(5) $$\mathrm{MCC}=\frac{\left(\mathrm{TP}\;\times \;\mathrm{TN})-(\mathrm{FP}\;\times \;\mathrm{FN}\right)}{\sqrt{\left(\mathrm{TP}\;+\;\mathrm{FP})(\mathrm{TP}\;+\;\mathrm{FN})(\mathrm{TN}\;+\;\mathrm{FP})(\mathrm{TN}\;+\;\mathrm{FN}\right)}}$$

where TP (true positives) denotes successfully identified toxicants; TN (true negatives) indicates correctly identified non-toxicants; FP (false positives) signifies non-toxicants that were incorrectly classified as toxicants; and FN (false negatives) represents toxicants that were wrongly predicted to be non-toxic. MCC values range between −1 and 1, with a perfect classification yielding a value of 1 and a random classification yielding a value of 0. Additionally, the area under the receiver operating characteristic curve (AUROC) and the area under the precision recall curve (AUPRC) were also used to determine classification capability.

### 2.6. Model Explainability

With the current pace of advancement of AI in drug development and related domains, there will be a growing demand for techniques that aid in the understanding of the complex machine learning models [29]. To address the explainability gap in several complex models and to enhance human understanding and decision-making, explainable AI (XAI) techniques have been emphasized. Explainability of supervised machine learning models is critically important in healthcare applications, especially in drug discovery, in addition to model performance [30,66]. The inherent black-box nature of machine learning methods such as neural networks and SVM algorithms may result in a lack of explainability. The explanation of machine learning predictions might be model-agnostic (independent) or model-specific. Additionally, model explanation methods can be global or local. Global model explanations can be generated using the ML model, trained on the training data, to provide insights regarding the internal workings of the complete model. However, instance explanations can be used to explain only a specific model prediction for a specific data sample [35].

The model-independent kernel SHAP approach was used in this work to explain the respiratory toxicity predictions. Lundberg and Lee invented the SHAP (SHapley Additive exPlanations) method to provide model transparency [36] for any machine learning algorithm. This approach was first developed to quantify the relevance of an individual player in a team. The goal of this theory was to distribute the overall reward among players based on their relative contributions to the game’s outcome. Shapley values were used to determine a reasonable and fair payoff for each player [67]. In the perspective of toxicity predictions, Shapley values were used to estimate the importance of descriptors (the magnitude of the impact) and also their direction (sign). The positive sign molecular descriptors contributed to the prediction of respiratory toxicants, whereas the negative sign descriptors contributed to the prediction of non-toxicants.

## 3. Results and Discussion

### 3.1. Data Distribution and Chemical Structural Diversity

**Figure 2:**
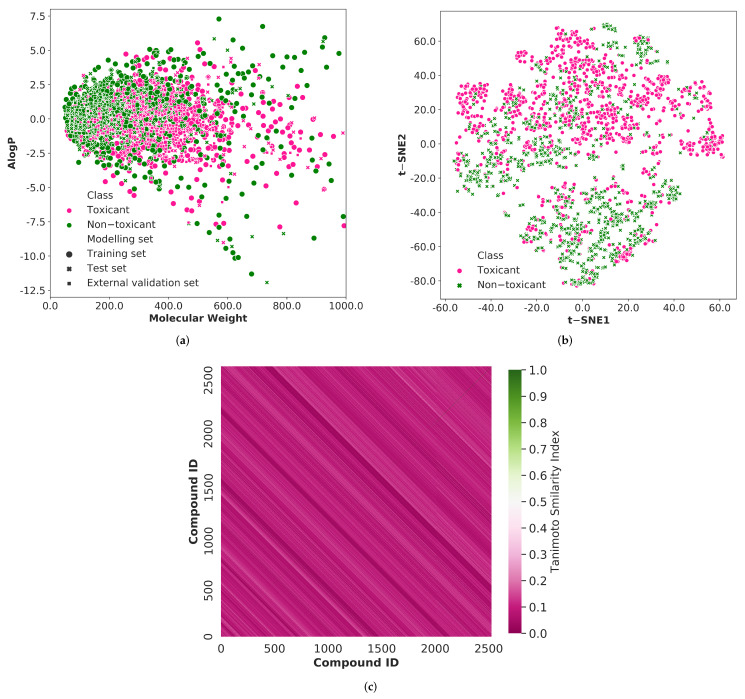
(**a**) The chemical spacedistribution of the respiratory toxicity training set (circle marker), test set (cross marker), and external validation set (square marker) defined by molecular weight and Ghose-Crippen LogKow (ALogP). The deep pink color indicates the respiratory toxic compounds and the green color indicates the respiratory non-toxic compounds. (**b**) The chemical space visualization using t-distributed stochastic neighbor embedding (t-SNE) using 50 principal components (represents 88% of the total variation) of the entire dataset. The deep pink color circle markers represent the respiratory toxic chemicals and the green color cross markers represent the respiratory non-toxic chemicals. (**c**) The heatmap of the Tanimotto similarity index of the compounds in the whole dataset. The pink color shows the low similarity index and green color shows the high similarity index of the compounds.

From the literature [28], we have gathered a total of 2527 chemical compounds associated with respiratory system toxicity. Among these chemicals, 1438 were respiratory toxicants, and 1089 were non-toxicants. The diversity of the chemical compounds utilized in training, testing, and external validation sets was analyzed in order to build a reliable classification model. As shown in Figure 2, we explored the chemical space of the whole dataset using molecular weight and Ghose-Crippen Logkow (ALogP). The molecular weight distribution is mostly between 50 and 800, while the ALogP variation is mostly between −8.5 and 5.0. It can be observed that the training set, test set, and external validation data are mostly covered by the same spatial distribution. Thus, the prediction results from the test set and external validation set can be used to evaluate the reliability of the prediction models built by the training set.

In addition, we used principal component analysis (PCA), one of the most widely used approaches, to evaluate the chemical space coverage of molecular descriptors across the entire 2527 molecules. However, the first two principal components account for only 38% of the total variation in the data. When the number of principal components was increased from 2 to 50, the overall variance of the data was 88%. Thus, another dimensionality reduction technique named t-distributed stochastic neighbor embedding (t-SNE) was applied with 50 principal components to obtain a reasonably accurate representation of the chemical space of the whole dataset (Figure 2).

To investigate more about the prediction model’s generalization ability, we analyzed the structural diversity of the compounds used in this study. The Morgan fingerprint is used to estimate the Tanimoto similarity index of the entire molecules, and the average similarity value is 0.082. The lower Tanimoto similarity index demonstrates the greater structural diversity of the compounds. The heatmap of the similarity index distribution is depicted in Figure 2. The heatmap is predominantly pink, implying that the compounds in the entire dataset exhibit significant structural diversity.

### 3.2. Selection of Important Chemical Descriptors

Molecular descriptors are properties of a molecule that have been determined experimentally or theoretically. More precisely, they are numerical representations of molecules’ physical, chemical, or topological properties that represent the knowledge of molecular structure and activity from a variety of perspectives. We have calculated a total of 1544 numbers of 1D and 2D PaDEL molecular descriptors for the chemical compounds used in this research to develop QSAR models for respiratory system toxicity prediction [45]. The majority of the computed descriptors are autocorrelation, topological, and electrotopological state descriptors. We have removed the features with empty and noisy values from the original descriptor set during the data preprocessing phase. We have normalized the descriptors using the min-max normalization technique before training the model on them. The low variance filtering feature selection method was used to automatically filter out nearly all zero-variance descriptors that do not contribute useful information to prediction. Additionally, we investigated the effects of various feature selection approaches on the selection of more informative features. To examine the effectiveness of the selected descriptors, these feature selection approaches were combined with an SVM classifier.

#### 3.2.1. Single Feature Selection Methods

**Figure 3:**
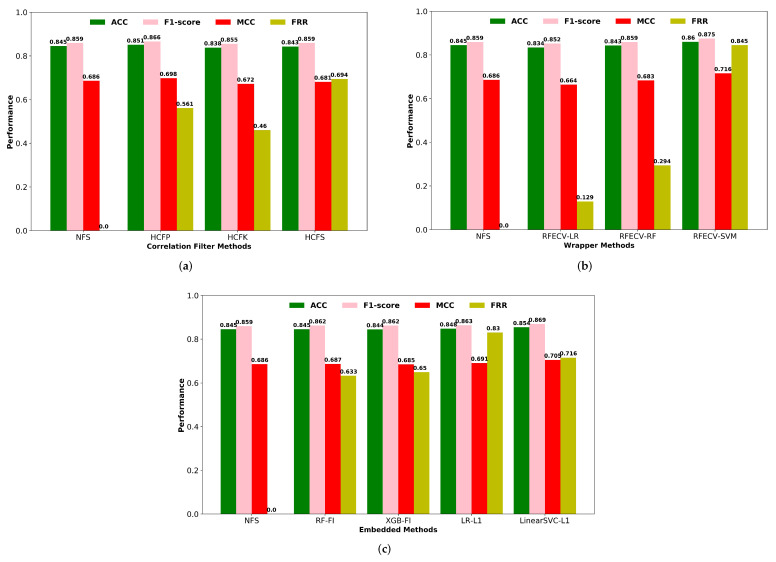
(**a**) Performance of correlation filters based feature selection methods. NFS: No feature selection; HCFP: High correlation filtering using Pearson’s correlation coefficient; HCFK: High correlation filtering using Kendall’s rank correlation coefficient; HCFS: High correlation filtering using Spearman’s rank correlation coefficient. (**b**) Wrapper methods. (**c**) Embedded Methods. NFS: No feature selection; RF-FI: Random Forest Classifier based feature importance; XGB-FI: XGB Classifier based feature importance; LR-L1: Logistic regression with L1-based feature selection; Linear SVC-L1: Linear SVC with L1-based feature selection.

We have compared the cross-validation performance of an optimized SVM classifier using descriptors obtained from several single feature selection methodologies, including filters, wrappers, and embedded methods. Filter-based feature selection techniques can reduce the computational time and resources required to train the model by discarding redundant and non-informative features from the high dimensional feature space. In filter methods, the multivariate correlation filtering methods were applied, including high correlation filtering with Pearson’s linear correlation coefficient (HCFP), Kendall’s rank correlation coefficient (HCFK), and Spearman’s rank correlation coefficient (HCFS). To reduce redundancy, we retained one of the descriptors with a mutual correlation greater than 90%. Furthermore, we discarded descriptors that had a very low correlation with the respiratory toxicity endpoint. The cross-validation performance of the correlation filter-based feature selection methods was compared in terms of F1-score, classification accuracy, MCC, and feature reduction rate (FRR) (Figure 3). The performance of descriptors selected using Pearson correlation coefficients outperforms other correlation-based filtering methods. This most widely used linear correlation method selects less than half of the descriptors from the useful descriptors set.

In contrast to the filter techniques, wrapper approaches use prediction accuracy to assess the relevance of a descriptor subset via an extensive search of the potential descriptors. An automatic feature selection approach in the form of a wrapper type RFECV was used in combination with LR, SVM, and RF algorithms to eliminate irrelevant features based on the cross-validation performance (Figure 3). The SVM-based RFECV technique outperformed the RF-based wrapper method in terms of prediction performance and also achieved the highest feature reduction rate of 84.5% of the initial descriptor set. Cross-validation accuracy and the number of descriptors selected by RFECV with LR, RF, and SVM algorithms are shown in Supplementary Figure S1.

In embedded tree-based feature selection, the RF classifier selects a few more significant descriptors with higher feature importance scores than the XGB classifier (Figure 3). However, the performance of the selected descriptors by RF and XGB had comparable performance in terms of prediction accuracy and F1-score metrics. For embedded methods, we also utilized the Linear SVC and LR models for Lasso regularization (L1) to eliminate unimportant features from the high-dimensional feature space. Lasso shrinks the coefficient of the less significant features to zero, effectively removing them from the original descriptor set. With a higher rate of feature reduction, the classification performance of the L1 regularization-based feature selection was higher than tree-based feature selection methods. According to Figure 3, the wrapper-based RFECV-SVM feature selection method achieved a high classification performance for the SVM classifier as well as the highest feature reduction rate of the single feature selection approaches used in this study. Supplementary Table S5 contains the number of descriptors selected by each single feature selection approach, along with a comparison of their performance.

#### 3.2.2. Hybrid Feature Selection Methods

**Figure 4:**
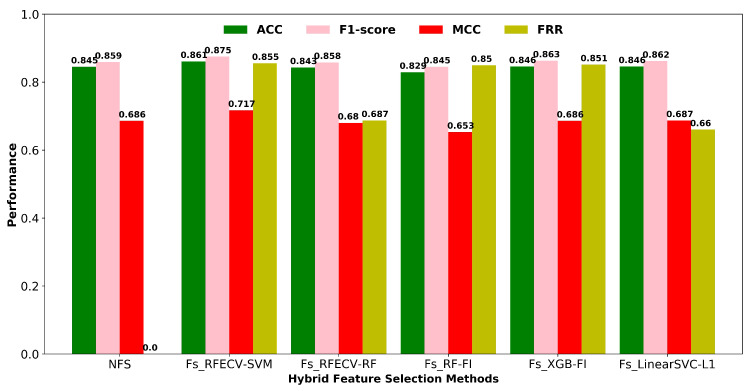
Hybrid feature selection methods with 2-stages. NFS: No feature selection; Stage 1: Fs (Low variance filtering and high correlation filtering using Pearson’s correlation coefficient); Stage 2: Wrapper based RFECV feature selection, Tree based embedded feature selection (FI-Feature Importance), and L1-based embedded feature selection.

While there are several methods for selecting features, including filters, wrappers, and embedded methods, each has its own set of advantages and disadvantages. As shown in Figure 4, we compared the classification performance of different two-stage hybrid feature selection (HFS) approaches using an SVM classifier, as well as the feature reduction rate for each method. In a high-dimensional feature space, wrapper-based feature selection is computationally expensive. Filtering techniques are much faster than wrapper approaches, so they are preferable for high-dimensional data. As a result, we employed a hybrid feature selection strategy in this work, which incorporates both filter and wrapper methods for selecting the best optimal descriptor subset. The first stage involved condensing the feature space using a zero variation filter and a high correlation filter based on Pearson linear correlation coefficients (correlation > 90%). The descriptors with a very low correlation with the respiratory toxicity endpoint are also eliminated based on the feature importance score (F-score). The second phase utilizes a wrapper–based RFECV to automatically find the optimal subset of descriptors from the filtered features to improve the classification performance. In terms of classification accuracy and MCC, the hybrid combination of filters and wrapper-based RFECV-SVM outperforms other HFS approaches depicted in Figure 4. The final number of descriptors selected using the best performing two-stage hybrid feature selection method is given in Supplementary Figure S2.

#### 3.2.3. Selected Descriptors Analysis

**Figure 5:**
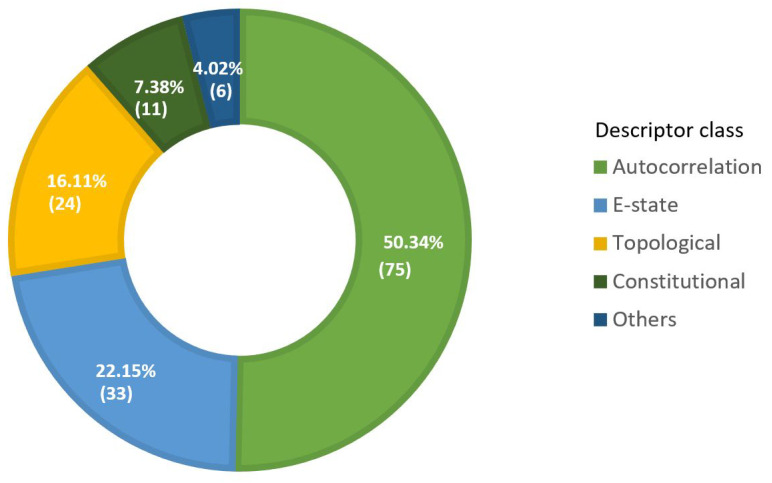
Donut chart to display descriptor class’s proportion (percentage contribution) in selected final descriptors subset (optimal set of descriptors).

The final set of descriptors selected for respiratory toxicity prediction includes descriptors from several classes, including autocorrelation, E-state, topological, Basak, and molecular-property descriptors (Figure 5). The donut chart also depicts the number of final descriptors in each class, along with their percentage contribution.

As illustrated in Figure 5, the majority of the selected descriptors belong to the autocorrelation, E-state, and topological descriptor classes. Table 3 lists the overall number of descriptors in these significant descriptor classes, as well as the corresponding descriptor types. Autocorrelation descriptors encode both the molecular structure and physicochemical properties of a molecule [68]. Autocorrelations are calculated by Geary (GATS), Moran (MATS), average Broto-Moreau (AATS), centered Broto-Moreau (ATSC), and average centered Broto-Moreau (AATSC) algorithms from lag 1 to lag 8 for various weighting schemes [69]. The term “lag” refers to the topological distance between two atoms. The lag parameter can take on any value in the range [0, 1, 2, 3, 4, 5, 6, 7, and 8]. The weight can be specified in terms of m (relative atomic mass), p (polarizability), e (Sanderson electronegativity), I (ionization potential), c (charges), and v (Van der Waals volume). For example, MATS2i is a Moran autocorrelation descriptor of lag 2 that is weighted by first ionization potential, and ATSC4e is a centered Broto-Moreau autocorrelation descriptor of lag 4 that is weighted by Sanderson electronegativities.

Electrotopological state (E-state) descriptors are used to describe a molecule’s molecular structure utilizing both electronic and topological properties [70]. There are thirty atom type electrotopological state descriptors in the selected optimal set of descriptors, e.g., minHother, maxHCsats. Electrotopological intrinsic states (E-state numbers) and E-state Indices (E-state contributions) are atomic-type electrotopological descriptors in which each skeletal atom or group is allocated an intrinsic state value. Several extended topological chemical atom descriptors (ETA), molecular distance-edge (MDE) descriptors, and mean topological charge index (JGI) descriptors are also included in the list of descriptors selected for modeling respiratory toxicity prediction [71,72]. The description of all the selected descriptors and their corresponding descriptor types can be found in Supplementary Table S6.

### 3.3. Prediction Performance of the Classification Models

We have employed hybrid feature selection methods to identify the optimal descriptors for 1869 compounds in the training dataset, which included 1043 respiratory toxicants and 826 non-toxic respiratory compounds. After selecting informative and significant representative molecular descriptors, various machine learning approaches were used to construct classification models of chemical-induced respiratory toxicity, including support vector machine (SVM), multi-layer perceptron (MLP), extreme gradient boosting (XGB), random forest (RF), logistic regression (LR), adaptive boosting decision tree (ABDT), k-nearest neighbors (KNN), and Naive Bayes (NB). In this study, the most extensively used data partitioning technique, tenfold cross-validation, is employed to efficiently utilize the training dataset to develop a more generalized model. The training dataset is randomly divided into 10 independent folds, nine of which are used to train the model and one of which is used to evaluate performance. The cross-validation procedure is then performed ten times, with each of the ten folds serving as validation data exactly once, and the results can be averaged to obtain the final prediction.

**Figure 6:**
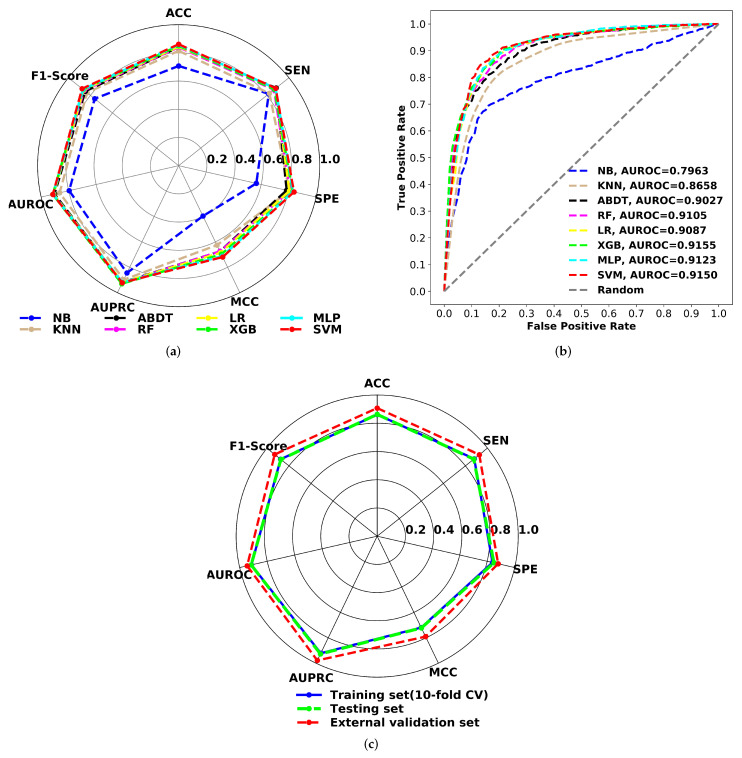
(**a**) Radar chart for 10-fold cross-validation performance comparison of ML models. (**b**) AUROC plots comparison for of ML models using 10-fold cross-validation. (**c**) Radar chart for comparing the proposed SVM model performance on respiratory toxicity training (10-fold CV), test and external validation data.

As shown in the radar chart (Figure 6), the performance of all optimized models was compared using internal validations for the training set. The structure of the plot can also be used to describe the quality of the models. A circle in the shape of the entire plot would represent a maximum score on all performance measures. The accuracy, specificity, sensitivity, F1-score, Matthews correlation coefficient, AUROC, AUPRC, and confusion matrix statistical values of all trained models have been reported in Supplementary Table S7. The plot clearly shows that the Naive Bayes classifier performs poorly across all metrics. The next-worst performing model is the KNN classification method. Except for these two models, all others have a model accuracy of over 84%, specificity of more than 78%, sensitivity greater than 86%, F1 score greater than 85%, MCC higher than 0.65, and an area under the ROC and PRC curves greater than 90%. The SVM model outperforms all other classification models in terms of ACC (0.862), MCC (0.720), and F1-score (0.876) for 10-fold cross-validation. Additionally, this model has the highest sensitivity (87.99%) and specificity (83.85%). Taking the ACC, MCC, and F1-score into account, the classification performance of all models was ranked from highest to lowest as SVM > MLP > XGB > LR > ABDT > RF > KNN > NB.

In Figure 6, the receiver operating characteristic (ROC) curves for all models were compared to the 10-fold CV. According to the AUROC scores, the SVM (0.9150) model performs comparably to the XGB model (0.9155). It is worth mentioning that the overall prediction performance of SVM is better than that of the MLP classifier and the XGB model (except AUROC and AUPRC by a very small margin of 0.1%) and also significantly better than that of the other reported models. MCC is a good predictor of binary classification performance for unbalanced datasets [73,74]. Based on the MCC and other performance metrics, the SVM model was proposed to develop classification models for predicting chemical-induced respiratory toxicity.

A test set of 465 compounds was used to evaluate the robustness and prediction power of the proposed classification model. The proposed model predicts 227 toxicants correctly out of 259; the sensitivity was 87.6%; and it also predicts 175 non-toxicants correctly out of 206, and the specificity was 84.9%. The F1-score, MCC, AUROC, and AUPRC scores for the test set are 0.874, 0.722, 0.916, and 0.927, respectively. When the radar plots are studied (Figure 6), it is clear that the proposed SVM model’s results for 10-fold cross-validation and the test set were virtually identical. The greater the shape of the test set, the more accurate the model. To assess the model’s generalizability, the model’s performance was evaluated against another external validation dataset of 193 chemical compounds. The suggested model reliably predicted 126 toxicants out of 136, with a sensitivity of 92.6%, and 50 non-toxicants out of 57, with a specificity of 87.9%.

As displayed in Figure 2, the chemical space distribution of the compounds in the model’s external validation set is more condensed than that of the test set, and they are primarily concentrated in the same area as the compounds in the training set. It indicates that the external validation set’s chemical structure is more comparable to that of the training set, implying that the model will predict them more accurately. The overall prediction results of the external validation set reveal that the proposed respiratory toxicity prediction model performs well across a variety of datasets. Additionally, our model’s predictions outperform earlier findings on chemical-induced respiratory toxicity (Table 4).

### 3.4. Model Explainability

The explainability of the models is often contingent upon evaluating the contribution of independent features (descriptors) to predictions. Although complex non-linear machine learning models are difficult to interpret, they are frequently utilized in molecular activity prediction and QSAR research. As a result, agnostic approaches for estimating the importance of features regardless of model complexity are essentially needed. To meet these demands, the SHAP technique was developed and validated by examining class label predictions of chemical compounds using a variety of machine learning algorithms.

#### 3.4.1. Global Feature Explanation

The main purpose of this work is to investigate the significant molecular descriptors that influence the classification model to provide accurate predictions of chemical-induced respiratory toxicity. According to the prediction performance of the optimized SVM model described previously, the optimal descriptors selected are capable of classifying the majority of respiratory toxicants from respiratory non-toxicants. This part focuses on identifying the most significant descriptors to confirm the black-box model’s reliability and improve its interpretability. The kernel SHAP approach is used to further explore the impact of selected molecular descriptors on the prediction of the proposed SVM model for chemical-induced respiratory toxicity.

**Figure 7:**
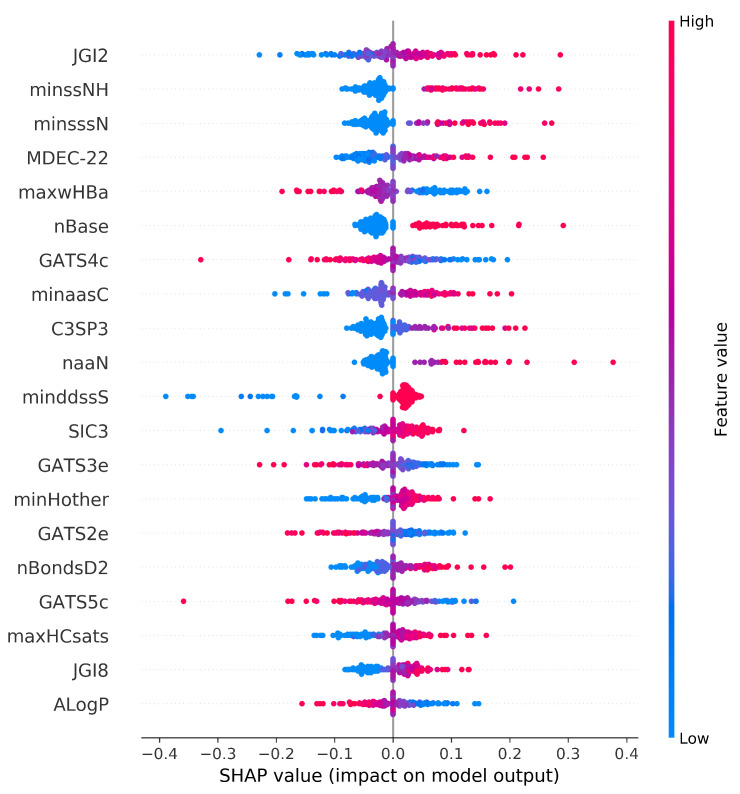
SHAP summary plot. Visualization of the Shapley values and molecular descriptor values in the SVM model. Each point is associated with a Shapley value for a single compound and descriptor. The color of the points is determined by their descriptor values, while the horizontal axis displays the Shapley values calculated. The vertical axis depicts both the descriptors and their distribution, arranged by their mean absolute Shapley values. The model’s most important descriptor is at the very top of the plot.

The SHAP summary plot shows the positive and negative relationships of the top twenty modeling descriptors with the respiratory toxicity class (Figure 7). The horizontal axis shows the actual SHAP values, representing the impact that the descriptors had on the model’s output. Descriptors are arranged in descending order along the vertical axis based on their importance. The most important descriptor, JGI2, is a two-ordered mean topological charge descriptor and has a positive impact on respiratory toxicity prediction. Atom type electrotopological state descriptor minssNH and molecular distance-edge descriptor MDEC-22 are the next most important descriptors that are positively correlated with respiratory toxicity. The description of the top ten important descriptors from the summary plot has been given in Table 5.

It is important to mention that when the top twenty selected molecular descriptors from the SHAP summary plot are studied, the majority of the electrotopological state atom types and topological descriptors contribute positively to the respiratory toxicity prediction. Likewise, the majority of Geary autocorrelation descriptors had a negative contribution towards the prediction. The most relevant descriptors reveal that electronic and structural descriptors are important for predicting chemical compounds’ respiratory toxicity. It is worth noting that E-state descriptors are capable of efficiently extracting structural information related to the toxicity of chemical compounds.

#### 3.4.2. Local Feature Explanation

In addition to global explanations, the SHAP values for each instance can be investigated to find the impact of each modeling descriptor on the model prediction output. Local explanations generated using the SHAP force plot explain how many features work together to push the model’s output from the base value to the predicted value. The average of all Shapley values is used as the base value. Each Shapley value is marked with an arrow throughout the plot, indicating whether to increase or decrease the prediction. The descriptors that contribute to a high prediction value are highlighted in red, and those that contribute to a low prediction value are highlighted in blue.

**Figure 8:**
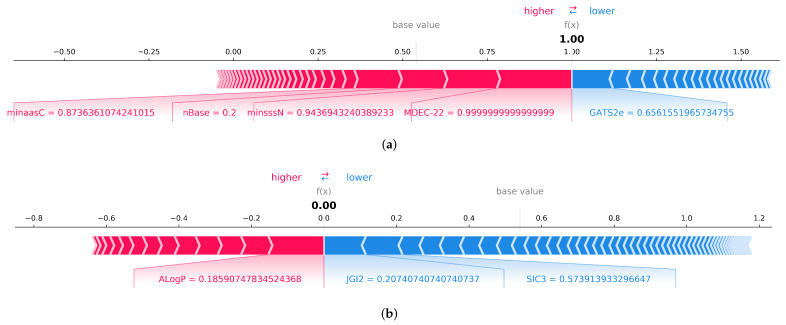
(**a**) Local explanation using SHAP force plot to explain a specific instance where the respiratory toxicity prediction is 1, and shows descriptors contributing towards the decision. (**b**) Local explanation using SHAP force plot to explain a specific instance where the respiratory toxicity prediction is 0.

The SHAP force plot (Figure 8) illustrates the impact of each descriptor on the model output to predict a specific compound as a respiratory toxicant or non-toxicant. The impact of each descriptor is proportionate to its bar length. In the given first example, the descriptors MDEC-22, minssN, nBase, and minaasC can enhance model output, whereas the autocorrelation descriptor GATS2e can decrease prediction. MDEC-22, a topological descriptor, has the greatest impact on the model, followed by E-state descriptors such as minsssN and minaasC. As shown in the second example, the ALogP descriptor closest to the diving border has a greater effect on negative prediction. The negative prediction score is lowered by the topological descriptor JGI2 and the structural information content descriptor SIC3. As a result, descriptors of topological, E-state, and structural information content can be considered the primary contributors in predicting respiratory toxicity.

## 4. Conclusions

In this research, a large dataset of diverse chemical compounds was used to develop prediction models for chemical-induced respiratory toxicity. Numerous feature selection methods were investigated, and a hybrid approach combining the best correlation filter and the RFECV wrapper technique was used to determine the most significant molecular descriptors for effective modeling. Then, eight machine learning classifiers for respiratory toxicity were constructed and validated using 10-fold cross-validation. The optimized SVM classifier performed better than the other classifiers. The proposed SVM model achieves an MCC of 0.722 and a prediction accuracy of 86.2% for the test set, and an external validation set was used to verify the model’s generalizability for different compounds. In addition, the SHAP explanation technique was explored to provide more relevant explanations that enhance model prediction transparency and also prioritize the significance of key modeling descriptors affecting the prediction results. The SHAP results revealed that the majority of E-state and topological descriptors have a positive impact on the prediction of respiratory toxicity. We believe that the explainable SVM classification model could be employed in pharmaceutical research as a tool for predicting and screening potential respiratory toxic chemicals. In the future, different global and local explanation techniques can be used to better understand the respiratory toxicity prediction models.

## Figures and Tables

**Table 1:** The number of compounds associated with respiratory toxicity in the modeling datasets.

Datasets	Toxicants	Non-Toxicants	Total
Training set	1043	826	1869
Test set	259	206	465
External validation set	136	57	193
Total	3772	1089	2527

**Table 2:** The list of molecular descriptors.

Descriptor Class	Dimension	Number of Descriptors
Constitutional descriptors	1	120
Autocorrelation descriptors	2	346
Basak descriptors	2	42
BCUT descriptors	2	6
Burden descriptors	2	96
Connectivity descriptors	2	56
E-state descriptors	2	489
Kappa descriptors	2	3
Molecular property descriptors	2	15
Quantum chemical descriptors	2	6
Topological descriptors	2	265

**Table 3:** The total number of descriptors in each significant descriptor class, with associated descriptor category.

Descriptor Class	Count	Descriptor Category	Count
Autocorrelation	75	Geary Autocorrelation Descriptor	25
		Moran Autocorrelation Descriptor	13
		Centered Broto-Moreau Autocorrelation Descriptor	13
		Average Broto-Moreau Autocorrelation Descriptor	12
		Average centered Broto-Moreau Autocorrelation Descriptor	12
E-state	33	Electro topological State Atom Type Descriptor	30
		Detour Matrix Descriptor	3
Topological	24	Extended Topo chemical Atom Descriptor	8
		Molecular Distance-Edge Descriptor	7
		Topological Charge Descriptor	6
		Others	3

**Table 4:** Comparison with existing classifiers.

No.	Model Name	No. of Compounds	Test Method	ACC	SPE	SEN	MCC
1	XGBoost [26]	468	Test set	0.826	0.832	0.822	0.644
2	NB-2 [27]	248	Test set	0.844	0.853	0.835	0.684
3	PubchemFP-RF [28]	1869	10-fold CV	0.840	0.805	0.868	0.675
		467	Test set	0.859	0.825	0.885	0.713
4	Proposed Model	1869	10-fold CV	0.862	0.838	0.879	0.717
		465	Test set	0.862	0.849	0.876	0.722

**Table 5:** Details of the top ten important descriptors that contribute to the prediction of respiratory toxicity.

Descriptor Name	Descriptor Class	Description	Impact
JGI2	Topological descriptors	Mean topological charge index of order 2	Positive
minssNH	E-state descriptors	Minimum atom-type E-State: -NH-	Positive
MDEC-22	Topological descriptors	Molecular distance edge between all secondary carbons	Positive
minsssN	E-state descriptors	Minimum atom-type E-State: >N-	Positive
nBase	Constitutional descriptors	Number of basic groups.	Positive
maxwHBa	E-state descriptors	Maximum E-States for weak Hydrogen Bond acceptors	Negative
GATS4c	Autocorrelation descriptors	Geary autocorrelation - lag 4/weighted by charges	Negative
minaasC	E-state descriptors	Minimum atom-type E-State: :C:-	Positive
C3SP3	Topological descriptors	Singly bound carbon bound to three other carbons	Positive
minddssS	E-state descriptors	Minimum atom-type E-State: >S==	Positive

## Data Availability

Not applicable.

## Supplementary Materials

The following supporting information can be downloaded at: https://www.mdpi.com/article/10.3390/pharmaceutics14040832/s1, Table S1: Training set compounds with molecular descriptors information.; Table S2: Testing set compounds with molecular descriptors information; Table S3: External validation set compounds with molecular descriptors information; Table S4: The optimal parameter values of the machine learning models; Table S5: The performance of various single and hybrid feature selection methods; Table S6: The details of the selected optimal descriptors; Table S7: The performance of machine learning classifiers using the optimal descriptors.

## References

[1] Vo A.H., Van Vleet T.R., Gupta R.R., Liguori M.J., Rao M.S. (2019). An overview of machine learning and big data for drug toxicity evaluation. Chem. Res. Toxicol..

[2] Basile A.O., Yahi A., Tatonetti N.P. (2019). Artificial intelligence for drug toxicity and safety. Trends Pharmacol. Sci..

[3] Cai M.C., Xu Q., Pan Y.J., Pan W., Ji N., Li Y.B., Jin H.J., Liu K., Ji Z.L. (2015). ADReCS: An ontology database for aiding standardization and hierarchical classification of adverse drug reaction terms. Nucleic Acids Res..

[4] Siramshetty V.B., Nickel J., Omieczynski C., Gohlke B.O., Drwal M.N., Preissner R. (2016). WITHDRAWN—A resource for withdrawn and discontinued drugs. Nucleic Acids Res..

[5] Valverde-Monge M., Fernández-Nieto M., López V.B., Rodrigo-Muñoz J.M., Cañas J.A., Sastre B., Del Potro M.G., De las Heras M., Del Pozo V., Sastre J. (2019). Novel causes of drug-induced occupational asthma. J. Allergy Clin. Immunol. Pract..

[6] Skeoch S., Weatherley N., Swift A.J., Oldroyd A., Johns C., Hayton C., Giollo A., Wild J.M., Waterton J.C., Buch M. (2018). Drug-induced interstitial lung disease: A systematic review. J. Clin. Med..

[7] Bartal C., Sagy I., Barski L. (2018). Drug-induced eosinophilic pneumonia: A review of 196 case reports. Medicine.

[8] Varghese M., Glaum M., Lockey R. (2010). Drug-induced rhinitis. Clin. Exp. Allergy.

[9] Schwaiblmair M., Behr W., Haeckel T., Märkl B., Foerg W., Berghaus T. (2012). Drug induced interstitial lung disease. Open Respir. Med. J..

[10] Chary A., Hennen J., Klein S.G., Serchi T., Gutleb A.C., Blömeke B. (2018). Respiratory sensitization: Toxicological point of view on the available assays. Arch. Toxicol..

[11] Matsuno O. (2012). Drug-induced interstitial lung disease: Mechanisms and best diagnostic approaches. Respir. Res..

[12] Cooper J.A.D., White D.A., Matthay R.A. (1986). Drug-induced pulmonary disease: Part 1: Cytotoxic drugs. Am. Rev. Respir. Dis..

[13] Rossi S.E., Erasmus J.J., McAdams H.P., Sporn T.A., Goodman P.C. (2000). Pulmonary drug toxicity: Radiologic and pathologic manifestations. Radiographics.

[14] Reinert T., Baldotto C.S.d.R., Nunes F.A.P., Scheliga A.A.d.S. (2013). Bleomycin-induced lung injury. J. Cancer Res..

[15] De Jonge M.E., Huitema A.D., Rodenhuis S., Beijnen J.H. (2005). Clinical pharmacokinetics of cyclophosphamide. Clin. Pharmacokinet..

[16] Madani Y., Mann B. (2012). Nitrofurantoin-induced lung disease and prophylaxis of urinary tract infections. Prim. Care Respir. J..

[17] Casey W., Jacobs A., Maull E., Matheson J., Clarke C., Lowit A. (2015). A new path forward: The interagency coordinating committee on the validation of alternative methods (ICCVAM) and national toxicology program’s interagency center for the evaluation of alternative toxicological methods (NICEATM). J. Am. Assoc. Lab. Anim. Sci..

[18] Rácz A., Bajusz D., Miranda-Quintana R.A., Héberger K. (2021). Machine learning models for classification tasks related to drug safety. Mol. Divers..

[19] Yang H., Sun L., Li W., Liu G., Tang Y. (2018). In silico prediction of chemical toxicity for drug design using machine learning methods and structural alerts. Front. Chem..

[20] Hua Y., Shi Y., Cui X., Li X. (2021). In silico prediction of chemical-induced hematotoxicity with machine learning and deep learning methods. Mol. Divers..

[21] Jiang C., Yang H., Di P., Li W., Tang Y., Liu G. (2019). In silico prediction of chemical reproductive toxicity using machine learning. J. Appl. Toxicol..

[22] Jaganathan K., Tayara H., Chong K.T. (2021). Prediction of Drug-Induced Liver Toxicity Using SVM and Optimal Descriptor Sets. Int. J. Mol. Sci..

[23] Mekenyan O., Patlewicz G., Kuseva C., Popova I., Mehmed A., Kotov S., Zhechev T., Pavlov T., Temelkov S., Roberts D.W. (2014). A mechanistic approach to modeling respiratory sensitization. Chem. Res. Toxicol..

[24] Jarvis J., Seed M., Stocks S., Agius R. (2015). A refined QSAR model for prediction of chemical asthma hazard. Occup. Med..

[25] Seed M.J., Agius R.M. (2017). Progress with Structure–Activity Relationship modelling of occupational chemical respiratory sensitizers. Curr. Opin. Allergy Clin. Immunol..

[26] Lei T., Chen F., Liu H., Sun H., Kang Y., Li D., Li Y., Hou T. (2017). ADMET evaluation in drug discovery. Part 17: Development of quantitative and qualitative prediction models for chemical-induced respiratory toxicity. Mol. Pharm..

[27] Zhang H., Ma J.X., Liu C.T., Ren J.X., Ding L. (2018). Development and evaluation of in silico prediction model for drug-induced respiratory toxicity by using naïve Bayes classifier method. Food Chem. Toxicol..

[28] Wang Z., Zhao P., Zhang X., Xu X., Li W., Liu G., Tang Y. (2021). In silico prediction of chemical respiratory toxicity via machine learning. Comput. Toxicol..

[29] Jiménez-Luna J., Grisoni F., Schneider G. (2020). Drug discovery with explainable artificial intelligence. Nat. Mach. Intell..

[30] Bannigan P., Aldeghi M., Bao Z., Häse F., Aspuru-Guzik A., Allen C. (2021). Machine learning directed drug formulation development. Adv. Drug Deliv. Rev..

[31] Rehman M.U., Tayara H., Chong K.T. (2021). DCNN-4mC: Densely connected neural network based N4-methylcytosine site prediction in multiple species. Comput. Struct. Biotechnol. J..

[32] Angelov P.P., Soares E.A., Jiang R., Arnold N.I., Atkinson P.M. (2021). Explainable artificial intelligence: An analytical review. Wiley Interdiscip. Rev. Data Min. Knowl. Discov..

[33] Rehman M.U., Akhtar S., Zakwan M., Mahmood M.H. (2022). Novel architecture with selected feature vector for effective classification of mitotic and non-mitotic cells in breast cancer histology images. Biomed. Signal Process. Control.

[34] Štrumbelj E., Kononenko I. (2014). Explaining prediction models and individual predictions with feature contributions. Knowl. Inf. Syst..

[35] Tjoa E., Guan C. (2020). A survey on explainable artificial intelligence (xai): Toward medical xai. IEEE Trans. Neural Netw. Learn. Syst..

[36] Lundberg S.M., Lee S.I. A unified approach to interpreting model predictions. Proceedings of the 31st International Conference on Neural Information Processing Systems.

[37] Rodríguez-Pérez R., Bajorath J. (2020). Interpretation of machine learning models using shapley values: Application to compound potency and multi-target activity predictions. J. Comput.-Aided Mol. Des..

[38] PNEUMOTOX. https://www.pneumotox.com/drug/index/.

[39] Hazardous Chemical Information System. http://hcis.safeworkaustralia.gov.au/.

[40] Dik S., Ezendam J., Cunningham A.R., Carrasquer C.A., van Loveren H., Rorije E. (2014). Evaluation of in silico models for the identification of respiratory sensitizers. Toxicol. Sci..

[41] Kuhn M., Letunic I., Jensen L.J., Bork P. (2016). The SIDER database of drugs and side effects. Nucleic Acids Res..

[42] Juan-Blanco T., Duran-Frigola M., Aloy P. (2015). IntSide: A web server for the chemical and biological examination of drug side effects. Bioinformatics.

[43] Alves V.M., Capuzzi S.J., Braga R.C., Borba J.V., Silva A.C., Luechtefeld T., Hartung T., Andrade C.H., Muratov E.N., Tropsha A. (2018). A perspective and a new integrated computational strategy for skin sensitization assessment. ACS Sustain. Chem. Eng..

[44] Todeschini R., Consonni V. (2009). Molecular Descriptors for Chemoinformatics: Volume I: Alphabetical Listing/Volume II: Appendices, References.

[45] Dong J., Cao D.S., Miao H.Y., Liu S., Deng B.C., Yun Y.H., Wang N.N., Lu A.P., Zeng W.B., Chen A.F. (2015). ChemDes: An integrated web-based platform for molecular descriptor and fingerprint computation. J. Cheminform..

[46] Guyon I., Elisseeff A. (2003). An introduction to variable and feature selection. J. Mach. Learn. Res..

[47] Tax D., Duin R. (2000). Feature scaling in support vector data descriptions. Learn. Imbalanced Datasets.

[48] Bollegala D. (2017). Dynamic feature scaling for online learning of binary classifiers. Knowl.-Based Syst..

[49] Bommert A., Sun X., Bischl B., Rahnenführer J., Lang M. (2020). Benchmark for filter methods for feature selection in high-dimensional classification data. Comput. Stat. Data Anal..

[50] Bolón-Canedo V., Sánchez-Marono N., Alonso-Betanzos A., Benítez J.M., Herrera F. (2014). A review of microarray datasets and applied feature selection methods. Inf. Sci..

[51] Bolboaca S.D., Jäntschi L. (2006). Pearson versus Spearman, Kendall’s tau correlation analysis on structure-activity relationships of biologic active compounds. Leonardo J. Sci..

[52] Khanal J., Lim D.Y., Tayara H., Chong K.T. (2021). i6ma-stack: A stacking ensemble-based computational prediction of dna n6-methyladenine (6ma) sites in the rosaceae genome. Genomics.

[53] Zhang X., Wang J., Gao Y. (2019). A hybrid short-term electricity price forecasting framework: Cuckoo search-based feature selection with singular spectrum analysis and SVM. Energy Econ..

[54] Boser B.E., Guyon I.M., Vapnik V.N. A training algorithm for optimal margin classifiers. Proceedings of the Fifth Annual Workshop on Computational Learning Theory.

[55] Murtagh F. (1991). Multilayer perceptrons for classification and regression. Neurocomputing.

[56] Chen T., He T., Benesty M., Khotilovich V., Tang Y., Cho H., Chen K. (2015). Xgboost: Extreme Gradient Boosting.

[57] Breiman L. (2001). Random forests. Mach. Learn..

[58] Kleinbaum D.G., Dietz K., Gail M., Klein M., Klein M. (2002). Logistic Regression.

[59] Freund Y., Mason L. The alternating decision tree learning algorithm. Proceedings of the Sixteenth International Conference on Machine Learning (ICML 1999).

[60] Cover T., Hart P. (1967). Nearest neighbor pattern classification. IEEE Trans. Inf. Theory.

[61] Rish I. An empirical study of the naive Bayes classifier. Proceedings of the IJCAI 2001 Workshop on Empirical Methods in Artificial Intelligence.

[62] Pedregosa F., Varoquaux G., Gramfort A., Michel V., Thirion B., Grisel O., Blondel M., Prettenhofer P., Weiss R., Dubourg V. (2011). Scikit-learn: Machine learning in Python. J. Mach. Learn. Res..

[63] Brownlee J. (2019). XGBoost with Python. Machine Learning Mastery.

[64] Czermiński R., Yasri A., Hartsough D. (2001). Use of support vector machine in pattern classification: Application to QSAR studies. Quant. Struct.-Act. Relatsh..

[65] Baldi P., Brunak S., Chauvin Y., Andersen C.A., Nielsen H. (2000). Assessing the accuracy of prediction algorithms for classification: An overview. Bioinformatics.

[66] Arrieta A.B., Díaz-Rodríguez N., Del Ser J., Bennetot A., Tabik S., Barbado A., García S., Gil-López S., Molina D., Benjamins R. (2020). Explainable Artificial Intelligence (XAI): Concepts, taxonomies, opportunities and challenges toward responsible AI. Inf. Fusion.

[67] Shapley L.S. (1953). A value for n-person games. Contributions to the Theory of Games, 2.

[68] Hollas B. (2003). An analysis of the autocorrelation descriptor for molecules. J. Math. Chem..

[69] Broto P., Moreau G., Vandycke C. (1984). Molecular structures: Perception, autocorrelation descriptor and sar studies: System of atomic contributions for the calculation of the n-octanol/water partition coefficients. Eur. J. Med. Chem..

[70] Hall L.H., Kier L.B. (1995). Electrotopological state indices for atom types: A novel combination of electronic, topological, and valence state information. J. Chem. Inf. Comput. Sci..

[71] Liu S., Cao C., Li Z. (1998). Approach to estimation and prediction for normal boiling point (NBP) of alkanes based on a novel molecular distance-edge (MDE) vector, *λ*. J. Chem. Inf. Comput. Sci..

[72] Galvez J., Garcia R., Salabert M., Soler R. (1994). Charge indexes. New topological descriptors. J. Chem. Inf. Comput. Sci..

[73] Chicco D., Tötsch N., Jurman G. (2021). The Matthews correlation coefficient (MCC) is more reliable than balanced accuracy, bookmaker informedness, and markedness in two-class confusion matrix evaluation. BioData Min..

[74] Abdelbaky I., Tayara H., Chong K.T. (2021). Prediction of kinase inhibitors binding modes with machine learning and reduced descriptor sets. Sci. Rep..

