# Supplementary material for: An Explainable Supervised Machine Learning Model for Predicting Respiratory Toxicity of Chemicals Using Optimal Molecular Descriptors
Source: Pharmaceutics. 2022 Apr 11;14(4):832. doi: 10.3390/pharmaceutics14040832 (PMC9028223; doi:10.3390/pharmaceutics14040832)
Supplement: Supplementary file 1 [file pharmaceutics-14-00832-s001.zip › pharmaceutics-1633938-supplementary/Supplementary_Files/Supplemental Information- Respiratory toxicity.pdf]

Supplementary Information

An Explainable Supervised Machine Learning Model for Predicting Respiratory Toxicity of Chemicals using Optimal Molecular Descriptors

Keerthana Jaganathan, Hilal Tayara* and Kil To Chong*

Content:

- **Table S1:** Training set compounds with molecular descriptors information.
- **Table S2:** Testing set compounds with molecular descriptors information.
- **Table S3:** External validation set compounds with molecular descriptors information.
- **Table S4:** The optimal parameter values of the machine learning models.
- **Table S5:** The performance of various single and hybrid feature selection methods.
- **Table S6:** The details of the selected optimal descriptors.
- **Table S7:** The performance of machine learning classifiers using the optimal descriptors.

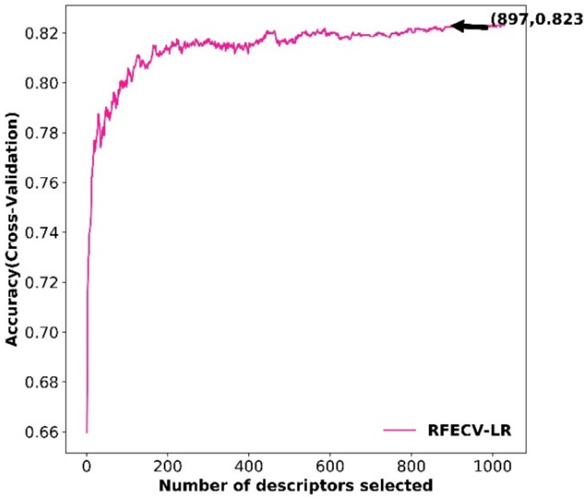

(a) Wrapper Method : RFECV-LR

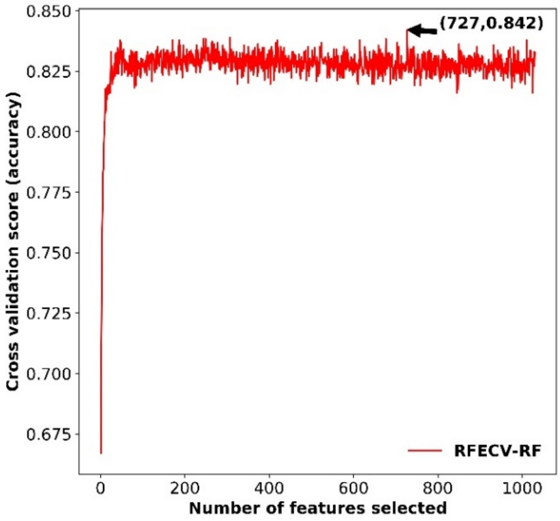

(b)Wrapper Method : RFECV-RF

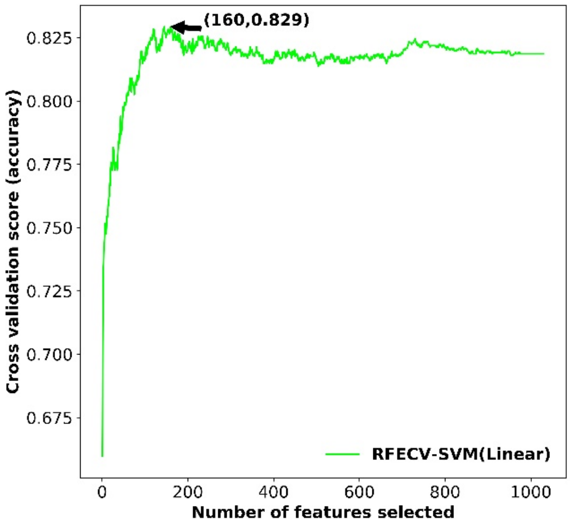

(c) Wrappers Method: RFEC V-SVM

Supplementary Figure S1. Single Feature Selection Methods(a-c)

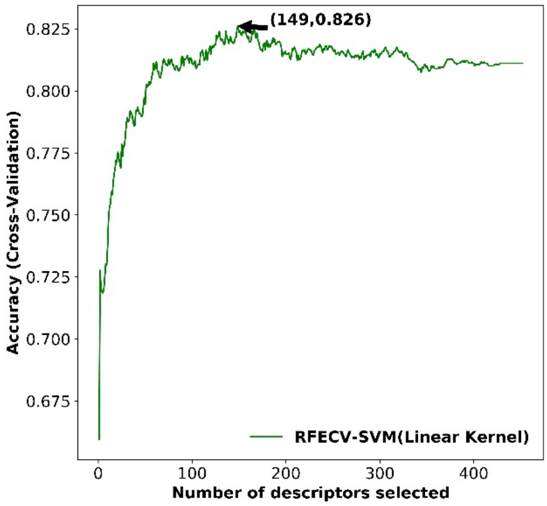

Supplementary Figure S2. Hybrid feature selection methods (Filter + Wrapper: Fs_RFECV-SVM)
